# Reversible and Fragile Watermarking for Medical Images

**DOI:** 10.1155/2018/3461382

**Published:** 2018-07-22

**Authors:** Atta-ur-Rahman, Kiran Sultan, Nahier Aldhafferi, Abdullah Alqahtani, Maqsood Mahmud

**Affiliations:** ^1^Department of Computer Science (CS), College of Computer Science and Information Technology (CCSIT), Imam Abdulrahman Bin Faisal University (IAU), P.O. Box 1982, Dammam, Saudi Arabia; ^2^Department of CIT, JCC, King Abdulaziz University, Jeddah, Saudi Arabia; ^3^Department of CIS, College of Computer Science and Information Technology (CCSIT), Imam Abdulrahman Bin Faisal University (IAU), P.O. Box 1982, Dammam, Saudi Arabia; ^4^Department of MIS, College of Business Administraction, Imam Abdulrahman Bin Faisal University (IAU), P.O. Box 1982, Dammam, Saudi Arabia

## Abstract

A novel reversible digital watermarking technique for medical images to achieve high level of secrecy, tamper detection, and blind recovery of the original image is proposed. The technique selects some of the pixels from the host image using chaotic key for embedding a chaotically generated watermark. The rest of the pixels are converted to residues by using the Residue Number System (RNS). The chaotically selected pixels are represented by the polynomial. A primitive polynomial of degree four is chosen that divides the message polynomial and consequently the remainder is obtained. The obtained remainder is XORed with the watermark and appended along with the message. The decoder receives the appended message and divides it by the same primitive polynomial and calculates the remainder. The authenticity of watermark is done based on the remainder that is valid, if it is zero and invalid otherwise. On the other hand, residue is divided with a primitive polynomial of degree 3 and the obtained remainder is appended with residue. The secrecy of proposed system is considerably high. It will be almost impossible for the intruder to find out which pixels are watermarked and which are just residue. Moreover, the proposed system also ensures high security due to four keys used in chaotic map. Effectiveness of the scheme is validated through MATLAB simulations and comparison with a similar technique.

## 1. Introduction

Cryptography, watermarking, and steganography are technologies that are frequently being used to ensure the security, authentication, and privacy (hiding) of data, respectively, especially when it is transmitted over a public network [1–5]. In cryptography, the message is encrypted in such a way that it becomes incomprehensible. In watermarking, the message watermark (text, image) is embedded in the host data (image/file) in such a way that host remains imperceptible and can be authenticated later, whereas in steganography the message is embedded in a host without getting any attention of user other than intended. Transmission of an encrypted message may create suspense for an intruder, whereas this is not a case with a stego or watermarked message in a cover signal. Nevertheless, combination of these technologies can be used more protection [1].

Unlike the cryptography, the steganography and the watermarking benefits from the perceptual limitations of human audiovisual systems (HAVS), which fail to recognize difference between original and watermarked/stego-signals respectively [6]. Usually, in steganography the media files such as, image, audio, or video are used as host signals to hide the message. In general, using an image or video as steganography cover signal is more popular than the audio. This is because HVS is far less sensitive to noise in the signal than HAS [7, 8]. In [9], authors proposed a spread spectrum (SS) based invertible watermarking scheme for medical images. In this scheme, single chaotic map along with residue number systems was utilized to obtain the fragility and reversibility. In [10], authors proposed a reversible watermarking scheme for medical images using product codes and RNS. The technique provides good safeguard against various attacks due to the product codes as error correction codes but at the cost of a little enhanced complexity. In [11–13], authors proposed a robust watermarking scheme using product code and cubic product code respectively. These codes provide a high level of error correcting capability against a variety of attacks in the image watermarking. Compared to simple product code, cubic product codes provide more error correction capability because of 3D codes. Similarly, in [14, 15], authors proposed reversible watermarking schemes for the medical images. In [16], authors presented a fragile image watermarking technique using SVD characteristics for authentication. Akin to this, in [17], authors presented a perceptual hash algorithm for multispectral image authentication. Yu et al. presented a comprehensive review of recoverable and nonrecoverable semifragile watermarking techniques on medical images in spatial and transform domain [18].

In this paper, a reversible image watermarking for medical images using RNS, CRC and double chaotic key is proposed. The scheme is highly fragile against any kind of tampering and highly sensitive to any type of change in the initial condition of chaotic system. Rest of the paper is organized as follows.  Section 2 contains the detailed description of the system model being used for the sake of embedding and extracting the watermark into the host medical image in a step-by-step way.  Section 3 contains the simulation results after experiment of the proposed scheme and Section 4 concludes the paper.

## 2. Proposed System Model

**Figure 1:**
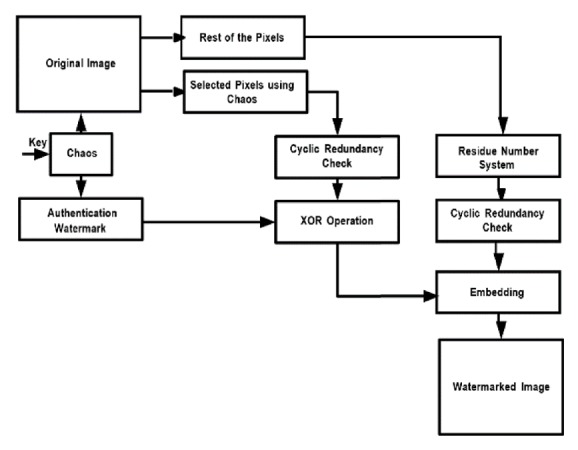
Schematic for watermark embedding.

**Figure 2:**
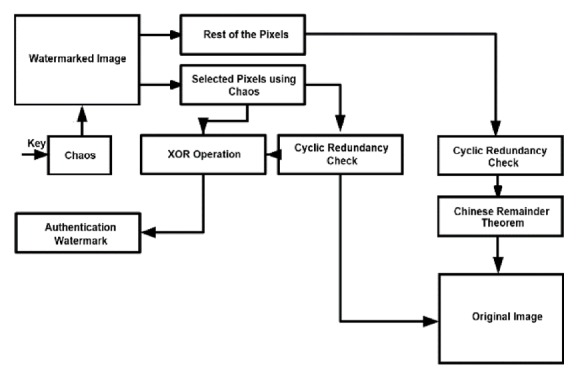
Schematic for watermark extraction.

The block diagram for watermark embedding is given in Figure 1. Block diagram for extraction of watermark & original image can be seen in Figure 2. Detailed steps of embedding procedure are as follows.

### 2.1. Watermark Embedding

- Preprocessing
- Generation of chaos based sequence to determine the location of pixels to be watermarked.
- Watermark embedding in these pixels.
- Finding residues of the rest of the pixels and appending CRC bits.

The details of these mentioned components are given subsequently.

#### 2.1.1. Preprocessing

Considering a gray-scale image of dimension MxN where M corresponds to rows and N corresponds to column, having pixels in intensity range [0,255]. The factors of the highest value 255 can have factors 15 and 17, respectively, that are relatively prime and correspond to moduli (15,17) of the host medical image. Consequently, a pixel having intensity between 0 and 255 can be divided by the two factors to obtain the corresponding moduli. The procedure is adapted from previous work given in [20, 21].

#### 2.1.2. Generation of Chaos

(i) Firstly, let us generate two separate binary chaotic sequences from the logistic map of (1). However, assume different initial conditions:
  (1) $$\begin{array}{c} A_{n+1}=rA_{n}\left(\;1-A_{n}\;\right) \end{array}$$
where 3.57 < *r* ≤ 4 and x_0_ belongs to (0,1).
(ii) Multiply the sequences by 8 and take its lower integer (ceil) to obtain the chaotic integers with each integer range from 1 to 8 as follows:
  (2) $$\begin{array}{c} A_{n+1}=ceil\left(\;A_{n+1}*8\;\right) \end{array}$$
For the sake of simplicity, the chaotic integers from two logistic maps are
  (3) P1=A1,A2,A3,…P2=B1,B2,B3,…
(iii) Add the chaotic integers and change them into sum sequences as
  (4) Q1=A1,A1+A2,A1+A2+A3,…Q2=B1,B1+B2,B1+B2+B3,…
For the sake of simplicity, the two sequences generated from (4) are
  (5) Q1=Q11,Q12+Q13,…,Q1pQ2=Q21,Q22+Q23,…,Q2q
where *Q*_1*p*_ ≤ *M* and *S*_2*q*_ ≤ *N*, with M and N representing the image's dimensions. For the chaotically chosen pixel pairs, [(*Q*_11_, *Q*_21_) + (*Q*_11_, *Q*_22_) ... (*Q*_11_, *Q*_2*q*_)] the watermark to be embedded is *A*_1_ and for the pixel pairs [(*Q*_12_, *Q*_21_) + (*Q*_12_, *Q*_22_) ... (*Q*_12_, *Q*_2*q*_)] the watermark to be embedded is *A*_2_ and so on.
(iv) Now the pixels to be watermarked have positions w.r.t the Cartesian product set *Q* = *Q*_1_*∗Q*_2_.

#### 2.1.3. Watermark Embedding

When the pixels to be watermarked are chosen using the chaotic key, the watermark embedding consists of the following steps.

(i) Choose the primitive polynomial *G*(*Y*) of degree 4 as
  (6) $$\begin{array}{c} G\left(\;Y\;\right)=Y^{4}+Y^{3}+1 \end{array}$$
(ii) Make the message polynomial *M*(*Y*) which is a pixel value in our case. Multiply *M*(*Y*) by *Y*^4^ and divide it by *G*(*Y*) to generate the remainder *R*(*X*) as
  (7) $$\begin{array}{c} Rem\left[\;\frac{X^{4}M\left(Y\right)}{G\left(Y\right)}\;\right]=R\left(\;Y\;\right) \end{array}$$
where *degree* (*R*(*Y*)) < *degree* (*G*(*Y*)).
(iii) Since the watermark for each pixel pair consists of four bits each and *R*(*Y*) is also consisted of four bits so R(Y) is XORed with corresponding watermark (A) as
  (8) $$\begin{array}{c} C\left(\;X\;\right)=R\left(\;X\;\right)⊕A \end{array}$$
(iv) Append *C*(*Y*) with *M*(*Y*) as
  (9) $$\begin{array}{c} T\left(\;Y\;\right)=\left[\;C\left(\;Y\;\right),M\left(\;Y\;\right)\;\right] \end{array}$$
where *T*(*Y*) is the original watermark pixel.

#### 2.1.4. Finding Residue and Appending CRC Bits

As we have seen, there is a set of pixels chosen in the image by a chaotic key in which the watermark is embedded. The complementary set of pixels undergoes the process of residue with CRC given below.

As explained above, our dynamic range is 0 to 254 and* m*_1_= 15,* m*_2_ =17. For any pixel, the residues (x_1_,x_2_) are obtained, where *x*_*i*_ = *X* mod *m*_*i*_. Since x_1_ ≤ 14 and x_2_ ≤ 16, x_1_ can be represented by four bits and x_2_ by five bits which makes a total of nine bits. Let us treat these nine bits as Residue Polynomial* Res*(*Y*) whose highest degree can be eight.

(i) For each residue, make its polynomial Res(*Y*) and choose the primitive polynomial *H*(*Y*) as
  (10) $$\begin{array}{c} H\left(\;Y\;\right)=Y^{3}+Y^{2}+1 \end{array}$$
(ii) Multiply Res(*X*) by X^3^ and divide it by *H*(*X*) to get the remainder Rem(*X*) as
  (11) $$\begin{array}{c} Rem\left[\;\frac{Y^{3}Res\left(Y\right)}{H\left(Y\right)}\;\right]=Rem\left(\;Y\;\right) \end{array}$$
(iii) Append *Rem*(*Y*) and *Res*(*Y*) as;
  (12) $$\begin{array}{c} R\left(\;Y\;\right)=Y^{3}Res\left(\;Y\;\right)+Rem\left(\;Y\;\right) \end{array}$$
*R*(*Y*) is at the most eleven-degree polynomial that represents 12 bits.

### 2.2. Watermark and Host Image Extraction

Watermark and original image extraction consists of following steps:

- Indicating the watermarked pixels using the chaotic key
- Watermark extraction and comparing
- Residues and CRC

#### 2.2.1. Indicating Watermark Pixels

With the knowledge of the chaotic key of encoding side, the watermarked pixels can be given as

(13) i  Q1=Q11,Q12+Q13,…,Q1pii  Q2=Q21,Q22+Q23,…,Q2q

#### 2.2.2. Watermark Extraction and Comparing

Extract *C*(*Y*) from *T*(*Y*) and the remaining part is *M*(*Y*) which are the original pixels.

Multiply *M*(*Y*) by *Y*^4^ and divide it by the known primitive polynomial *G*(*Y*) used in embedding side:

(14) $$\begin{array}{c} Rem\left[\;\frac{Y^{4}M\left(Y\right)}{G\left(XY\right)}\;\right]=R\left(\;Y\;\right) \end{array}$$

After that, to obtain the original watermark back, perform exclusive OR between *C*(*Y*) and *R*(*Y*).

(15) $$\begin{array}{c} W=C\left(\;Y\;\right)⊕R\left(\;Y\;\right) \end{array}$$

If *W* is the same watermark that means no pixel in the message *M*(*Y*) is tampered and vice versa.

#### 2.2.3. Residue and CRC

The set of pixels that are watermarked are chosen by the chaotic key while rest of them are residue. Each residue is divided by the known primitive polynomial *H*(*Y*) and the remainder is obtained as

(16) =RemRYHY=RemY3ResY+RemYHY=0

If the remainder is zero, that is an indication that none of the bits is corrupted and by utilizing (3), the original coefficients of image are recovered; otherwise, the image is tampered.

## 3. Simulation Results

To depict the viability of the proposed scheme, the simulations were conducted in MATLAB-8b. The parameters used in the simulation with their respective values are enlisted in Table 1.

### 3.1. Security Analysis

**Figure 3:**
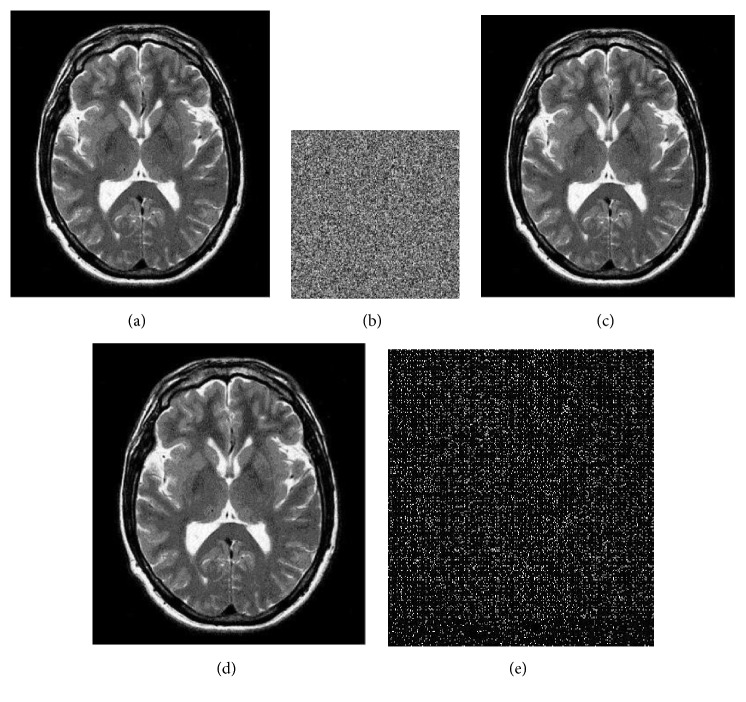
(a) Original MRI image; (b) chaotic watermark; (c) watermarked image. (d) Recovered image with exact initial conditions. (e) Recovered image with x(0) = 0.25000001, r = 3.58, and x(0) = 0.56, r = 3.57.

**Figure 4:**
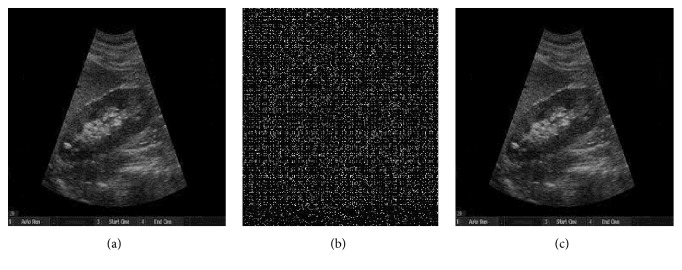
(a) Original MRI image; (b) recovered image with slightly modified initial conditions. (c) Recovered image with exact initial conditions.

Figure 3(a) shows the original host MRI image of size 348x314 for watermarking. The chaotic maps used are the logistic map given in (1) with initial conditions x(0) = 0.25, r = 3.58 and x(0) = 0.56, r = 3.57 respectively at embedding side and chaotic watermark pattern of size 55x55 is considered for experiment.  Figure 3(b) shows the chaotic watermark pattern.  Figure 3(c) shows the watermarked image.  Figure 3(d) shows the recovered image by using the exact initial conditions used on embedding side, that are, x(0) = 0.25 and r = 3.58 and x(0) = 0.56, r = 3.57 respectively.  Figure 3(e) shows the recovered image with slightly modified initial conditions, that are, x(0) = 0.25000001, r = 3.58 and x(0) = 0.56, r = 3.57. In Figure 3(d), original image is recovered exactly because same set of initial conditions are used to retrieve the image. As we can see in Figure 3(e) that when initial conditions are slightly modified, the original MRI image neither recovered back nor conceivable, which demonstrates the high secrecy of the proposed technique. Similarly, Figure 4 shows identical results with an ultrasound medical image. Figures 4(a), 4(b), and 4(c) show the original image, recovered image with slightly modified initial conditions, and recovered image with exact initial conditions respectively.

### 3.2. Fragility Analysis

**Figure 5:**
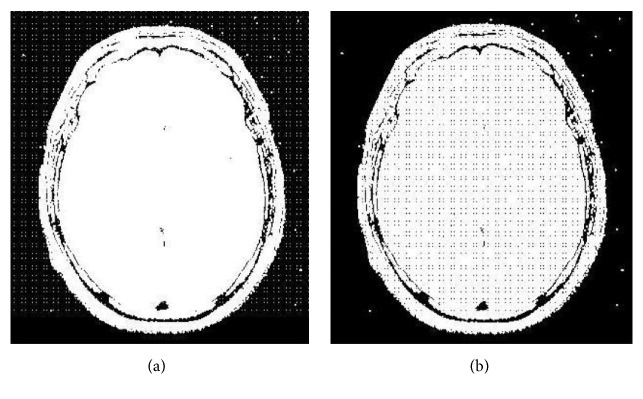
(a) Recovered image after AWGN attack. (b) Recovered image after Salt & Pepper noise attack.

In this section, the watermark is passed through various attacks and its fragility against those attacks is observed.  Figure 5(a) shows the recovered image after passing through additive white Gaussian noise (AWGN) attack of variance 0.02. Similarly, Figure 5(b) shows the recovered watermarked image after attacked from Salt & Pepper noise attack of variance 0.1. As it is apparent from the figures obtained that the images are not recognizable at all. Although this time exact initial conditions were applied for the sake of image reconstruction but, due to the added noise, the residues are not regenerated properly. That is why the outline boundary depicts that it was an MRI image but recovery is not possible. This shows that the scheme is highly fragile against any type of attack and subject to any type of tampering or change in the initial conditions, no matter how little that change is. The only way to obtain the exact image, the watermark, and error free delivery of the host image is to provide the exact initial conditions and untampered image at receiver side.

### 3.3. Imperceptibility Analysis

This is another figure of merit of the digital image watermarking. It is a measure of perceptual level of the watermarked image. That means, it must be noticed that image is watermarked. It is measured by peak signal to noise ratio (PSNR) of the image, given by the following [21]:

(17) PSNR=10 log10⁡2552MSEwhere  MSE=1MN∑i=0M−1 ∑j=0N−1fx,y−f′x,y2

The obtained PSNR value of the watermarked image using the proposed scheme is 72.98dB for MRI image and 69.22dB for ultrasound image which is a very good measure because PSNR level 30dB or higher is practically viable [19].

### 3.4. Comparison

Table 2 shows the comparison of the proposed scheme with a similar technique by [22].

## 4. Conclusion

This research focuses on a novel reversible watermarking scheme for medical images with blind recovery (no additional information needed) on receiver side. In the proposed scheme, the original host image is chaotically watermarked to provide maximum secrecy. The only thing we need for exact recovery is the knowledge of exact initial conditions as we have seen in the simulation results that slight modification in the initial conditions does not recover original image and the watermark which demonstrates the fragility and high security of the proposed scheme. Moreover, the proposed scheme exhibits a high level of imperceptibility which is inferred from obtained PSNR value of 72.98dB. In future, robustness characteristics can also be introduced in the scheme to make it hybrid or semifragile digital image watermarking scheme. Similarly, capacity analysis can also be performed.

## Figures and Tables

**Table 1:** Simulation parameters.

**Sr. No.**	**Parameter**	**Value**
1	Host medical image size	348x314
2	Watermark image size	55x55
3	Chaos initial conditions	x(0) = 0.25 r = 3.58 x(0) = 0.56, r = 3.57
4	Image type	MRI, Ultrasound
5	Attack type	AWGN, Salt & Pepper
6	Watermark type	Chaotically generated random sequence of grayscale pixels having intensity between 0-255

**Table 2:** Comparison summary.

**Sr. No.**	**Comparison parameter**	**Zain & Clarke [19]**	**Proposed scheme**
**1**	PSNR achieved	51.5dB	72.98dB

**2**	Security /authentication measures	Hash	CRC and Chaos

**3**	Embedding method and data	Plain LSB embedding with self-generated binary string	Residue with chaotically selected pixel embedding

**4**	Type of attacks	Adobe photoshop cloning	Salt & Pepper noise, AWGN

**5**	Tamper detection	Yes	Yes

**6**	Robustness of watermark	No	No

**7**	Host image	Fragile and recoverable only if there is no attack	Highly fragile and exactly recoverable without attack

**8**	Image type	Ultrasound	MRI, Ultrasound
